# Supplementary material for: Global impact of somatic structural variation on the cancer proteome
Source: Nat Commun. 2023 Sep 13;14:5637. doi: 10.1038/s41467-023-41374-8 (PMC10499989; doi:10.1038/s41467-023-41374-8)
Supplement: Supplementary file 1 — Supplementary Information [file 41467_2023_41374_MOESM1_ESM.pdf]

Supplementary Information

Zhang, Chen, Chandrashekar et al. “Global impact of somatic structural variation on the cancer proteome”

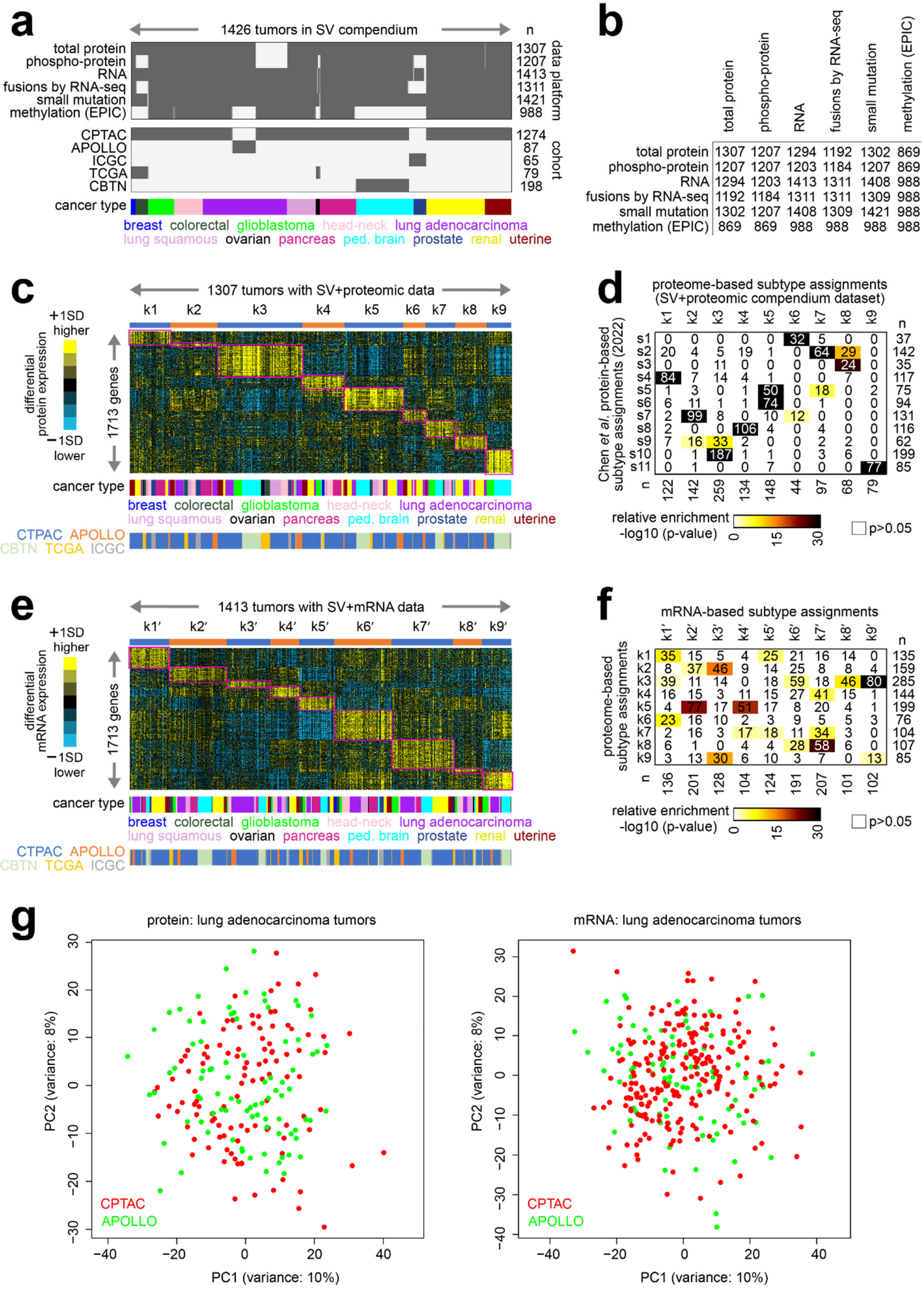
a
1426 tumors in SV compendium
n
total protein 1307
phospho-protein 1207
RNA 1413
fusions by RNA-seq 1311
small mutation 1421
methylation (EPIC) 988
data platform
CPTAC 1274
APOLLO 87
ICGC 65
TCGA 79
CBTN 198
cohort
cancer type
breast colorectal glioblastoma head-neck lung adenocarcinoma
lung squamous ovarian pancreas ped. brain prostate renal uterine
b
total protein | phospho-protein | RNA | fusions by RNA-seq | small mutation | methylation (EPIC)
total protein 1307 1207 1294 1192 1302 869
phospho-protein 1207 1207 1203 1184 1207 869
RNA 1294 1203 1413 1311 1408 988
fusions by RNA-seq 1192 1184 1311 1311 1309 988
small mutation 1302 1207 1408 1309 1421 988
methylation (EPIC) 869 869 988 988 988 988
c
1307 tumors with SV+proteomic data
k1 k2 k3 k4 k5 k6 k7 k8 k9
+1SD higher
differential protein expression
−1SD lower
1713 genes
cancer type
breast colorectal glioblastoma head-neck lung adenocarcinoma
lung squamous ovarian pancreas ped. brain prostate renal uterine
CTPAC APOLLO
CBTN TCGA ICGC
d
proteome-based subtype assignments
(SV+proteomic compendium dataset)
Chen et al, protein-based subtype assignments (2022)
k1 k2 k3 k4 k5 k6 k7 k8 k9 n
s1 0 0 0 0 0 32 5 0 0 37
s2 20 4 5 19 1 0 64 29 0 142
s3 0 0 11 0 0 0 0 24 0 35
s4 84 7 14 4 1 0 0 7 0 117
s5 1 3 0 1 50 0 18 0 2 75
s6 6 11 1 1 74 0 1 0 0 94
s7 2 99 8 0 10 12 0 0 0 131
s8 2 0 0 106 4 0 4 0 0 116
s9 7 16 33 2 0 0 2 2 0 62
s10 0 1 187 1 1 0 3 6 0 199
s11 0 1 0 0 7 0 0 0 77 85
n 122 142 259 134 148 44 97 68 79
relative enrichment
-log10 (p-value)
0 15 30
p>0.05
e
1413 tumors with SV+mRNA data
k1′ k2′ k3′ k4′ k5′ k6′ k7′ k8′ k9′
+1SD higher
differential mRNA expression
−1SD lower
1713 genes
cancer type
breast colorectal glioblastoma head-neck lung adenocarcinoma
lung squamous ovarian pancreas ped. brain prostate renal uterine
CTPAC APOLLO
CBTN TCGA ICGC
f
mRNA-based subtype assignments
proteome-based subtype assignments
k1′ k2′ k3′ k4′ k5′ k6′ k7′ k8′ k9′ n
k1 35 15 5 4 25 21 16 14 0 135
k2 8 37 46 9 14 25 8 8 4 159
k3 39 11 14 0 18 59 18 46 80 285
k4 16 15 3 11 15 27 41 15 1 144
k5 4 77 17 51 17 8 20 4 1 199
k6 23 16 10 2 3 9 5 5 3 76
k7 2 16 3 17 18 11 34 3 0 104
k8 6 1 0 4 4 28 58 6 0 107
k9 3 13 30 6 10 3 7 0 13 85
n 136 201 128 104 124 191 207 101 102
relative enrichment
-log10 (p-value)
0 15 30
p>0.05
g
protein: lung adenocarcinoma tumors
PC2 (variance: 8%)
PC1 (variance: 10%)
CPTAC
APOLLO
mRNA: lung adenocarcinoma tumors
PC2 (variance: 8%)
PC1 (variance: 10%)
CPTAC
APOLLO

Supplementary Figure 1. Additional information on the combined multi-omic and WGS datasets used in this study. (a) Data platforms, cohorts, and cancer types represented across the 1426-tumor cohort of combined SV and expression data (by protein or mRNA). **(b)** Overlap in samples represented between any two data platforms. See also Supplementary Dataset 1 for sample-level annotation by cohort and data platform availability. **(c)** The 1307 proteomic profiles do not cluster by cohort or cancer type. ConsensusClusterPlus[1] was used to call nine subtypes in the proteomic dataset using a set of 1713 genes for which all tumors had differential protein expression values. By design, because of our z-normalization of expression values within each dataset[2-7], the nine molecular subtypes cut across both cancer type and patient cohort. For the heat map, proteomic profiles are centered across protein features by the median within each profile. **(d)** Significance of overlap of the proteomic subtypes from part c (columns) with the previously identified pan-cancer subtypes from our proteogenomics study of 2002 tumors[4] (s1–s11, rows) is indicated. P-values by one-sided Fisher's exact test. Of the 1307 tumors in the combined SV and proteomics compendium dataset, 1093 were represented in the previous 2002-tumor study. **(e)** The 1413 transcriptomic profiles do not cluster by cohort or cancer type. ConsensusClusterPlus was used to call nine subtypes in the transcriptomic dataset using a set of 1713 genes from part c. Because of our z-normalization of expression values within each dataset, the nine molecular subtypes cut across both cancer type and patient cohort. **(f)** Significance of overlap of the mRNA-based subtypes from part e (columns) with the proteome-based subtypes from part c (rows). P-values by one-sided Fisher's exact test. **(g)** Proteomic and transcriptomic profiles of lung adenocarcinomas were represented in both CPTAC[8] and APOLLO[9] cohorts. Using the 1713 genes from part c, we carried out principal component analysis (PCA) for the combined set of lung adenocarcinomas using either the proteomic data (left) or the transcriptomic data (right). Out of 8574 proteins shared between the APOLLO lung and CPTAC lung proteomic datasets, just 6 were differential with $p<0.01$ (t-test).

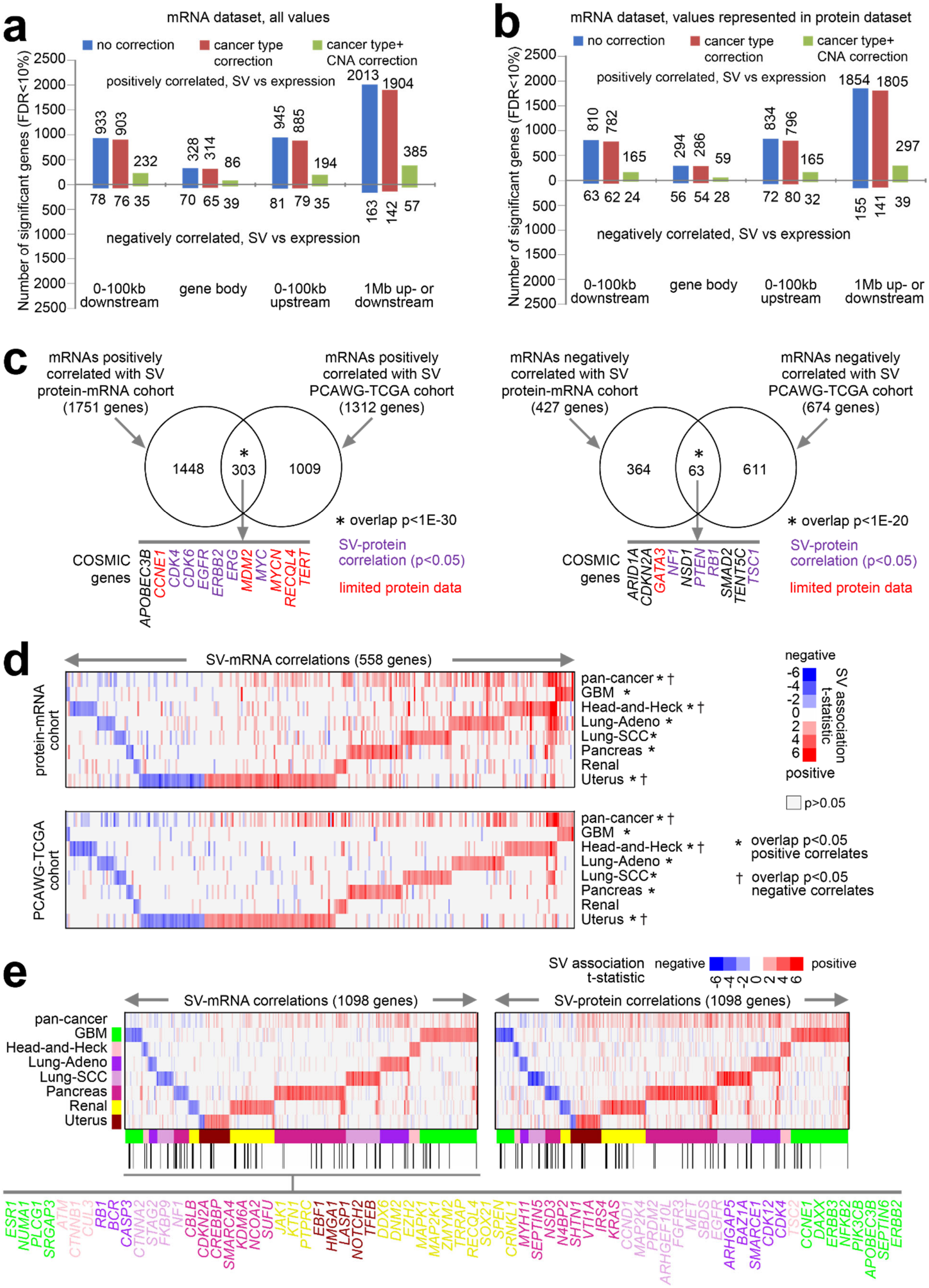
a
mRNA dataset, all values
no correction
cancer type correction
cancer type+ CNA correction
Number of significant genes (FDR<10%)
positively correlated, SV vs expression
negatively correlated, SV vs expression
933 903 232
328 314 86
945 885 194
2013 1904 385
78 76 35
70 65 39
81 79 35
163 142 57
0-100kb downstream
gene body
0-100kb upstream
1Mb up- or downstream
b
mRNA dataset, values represented in protein dataset
no correction
cancer type correction
cancer type+ CNA correction
Number of significant genes (FDR<10%)
positively correlated, SV vs expression
negatively correlated, SV vs expression
810 782 165
294 286 59
834 796 165
1854 1805 297
63 62 24
56 54 28
72 80 32
155 141 39
0-100kb downstream
gene body
0-100kb upstream
1Mb up- or downstream
c
mRNAs positively correlated with SV protein-mRNA cohort (1751 genes)
mRNAs positively correlated with SV PCAWG-TCGA cohort (1312 genes)
1448
* 303
1009
* overlap p<1E-30
COSMIC genes
APOBEC3B CCNE1 CDK4 CDK6 EGFR ERBB2 ERG MDM2 MYC MYCN RECQL4 TERT
SV-protein correlation (p<0.05)
limited protein data
mRNAs negatively correlated with SV protein-mRNA cohort (427 genes)
mRNAs negatively correlated with SV PCAWG-TCGA cohort (674 genes)
364
* 63
611
* overlap p<1E-20
COSMIC genes
ARID1A CDKN2A GATA3 NF1 NSD1 PTEN RB1 SMAD2 TENT5C TSC1
SV-protein correlation (p<0.05)
limited protein data
d
SV-mRNA correlations (558 genes)
protein-mRNA cohort
PCAWG-TCGA cohort
pan-cancer * †
GBM *
Head-and-Heck * †
Lung-Adeno *
Lung-SCC*
Pancreas *
Renal
Uterus * †
negative
-6 -4 -2 0 2 4 6
positive
SV association t-statistic
p>0.05
* overlap p<0.05 positive correlates
† overlap p<0.05 negative correlates
e
SV association t-statistic
negative
-6 -4 -2 0 2 4 6
positive
SV-mRNA correlations (1098 genes)
SV-protein correlations (1098 genes)
pan-cancer
GBM
Head-and-Heck
Lung-Adeno
Lung-SCC
Pancreas
Renal
Uterus
ESR1 NUMA1 PLCG1 SRGAP3 ATM CTNNB1 CUL3 RB1 BCR CASP3 CTNNA2 STAG2 FKBP9 NF1 CBLB CDKN2A CREBBP SMARCA4 KDM6A NCOA2 SUFU JAK1 KTN1 PTPRC EBF1 HMGA1 LASP1 NOTCH2 TFEB DDX6 DNM2 EZH2 MAPK1 MAP2K1 ZMYM2 TRRAP RECQL4 SOX21 SPEN CRNKL1 MYH11 SEPTIN5 NSD3 N4BP2 SHTN1 VTI1A IRS4 KRAS CCND1 MAP2K4 PRDM2 ARHGEF10L FGFR3 MET SBDS EGFR ARHGAP5 BAZ1A SMARCE1 CDK12 CDK4 TSC2 CCNE1 DAXX ERBB3 NFKB2 PIK3CB APOBEC3B SEPTIN6 ERBB2

Supplementary Figure 2. Additional information on genes with altered protein or mRNA expression associated with nearby somatic SV breakpoints. (a) For each of four genomic region windows in relation to genes (100kb upstream, 100kb downstream, within the gene, or 1MB upstream or downstream), the numbers of significant genes (using FDR <0.1) showing the association between mRNA expression and nearby SV breakpoint. Linear regression models evaluated significant associations without any correction for covariates, when correcting for cancer type, or for both cancer type and gene-level CNA. **(b)** Similar to part a, but using a filtered mRNA dataset, with any expression data values not represented in the protein dataset removed. The results for the filtered mRNA dataset would help assess how missing protein values contribute to disparate results between protein and mRNA datasets. **(c)** Venn diagrams representing the overlaps between the genes with mRNAs correlated (p<0.05) with SV breakpoint in our combined protein and mRNA compendium cohort (n=1413 tumors) and the genes correlated (p<0.05) with SV breakpoint in TCGA-ICGC cohort (n = 2334 tumors, 131 of which are represented in the 1413-tumor cohort)[10]. P values by one-sided Fisher's exact test. **(d)** Between the combined protein and mRNA compendium and TCGA-ICGC cohorts, 558 genes had mRNAs significantly associated with nearby SV breakpoints for one or more individual cancer types for both cohorts (p<0.05 for each, with consistent direction). For each cancer type, significant gene set overlap between positive or negative SV-expression correlates evaluated by one-sided Fisher's exact tests. **(e)** In our combined protein and mRNA compendium cohort, 1098 genes significantly associated with nearby SV breakpoints for one or more individual cancer types for both protein and mRNA (p<0.05 for both and p<0.01 for either, with consistent direction). Genes listed by name are COSMIC genes. Results for parts a and b are taken from the 10087 genes with protein values for at least 400 tumors (Figure 1b), while results for parts c-e are taken from the entire set of 15439 genes in the proteomics compendium dataset. For parts c-e, SV-expression associations are based on 1Mb region and correct for cancer type and CAN by linear modeling.

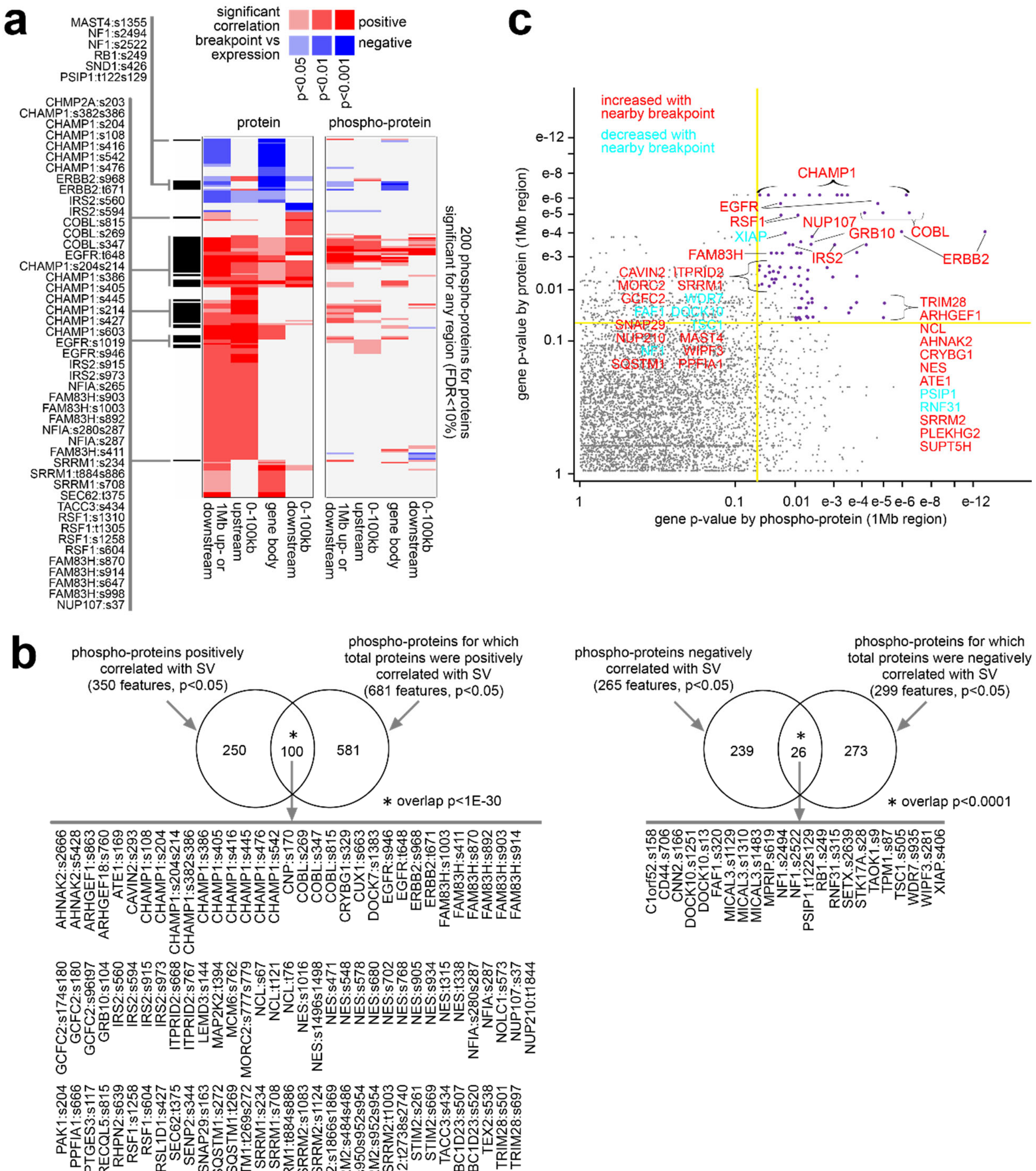
a
significant correlation breakpoint vs expression
positive
negative
p<0.05
p<0.01
p<0.001
protein
phospho-protein
200 phospho-proteins for proteins significant for any region (FDR<10%)
1Mb up- or downstream
0-100kb upstream
gene body
0-100kb downstream
MAST4:s1355
NF1:s2494
NF1:s2522
RB1:s249
SND1:s426
PSIP1:t122s129
CHMP2A:s203
CHAMP1:s382s386
CHAMP1:s204
CHAMP1:s108
CHAMP1:s416
CHAMP1:s542
CHAMP1:s476
ERBB2:s968
ERBB2:t671
IRS2:s560
IRS2:s594
COBL:s815
COBL:s269
COBL:s347
EGFR:t648
CHAMP1:s204s214
CHAMP1:s386
CHAMP1:s405
CHAMP1:s445
CHAMP1:s214
CHAMP1:s427
CHAMP1:s603
EGFR:s1019
EGFR:s946
IRS2:s915
IRS2:s973
NFIA:s265
FAM83H:s903
FAM83H:s1003
FAM83H:s892
NFIA:s280s287
NFIA:s287
FAM83H:s411
SRRM1:s234
SRRM1:t884s886
SRRM1:s708
SEC62:t375
TACC3:s434
RSF1:s1310
RSF1:t1305
RSF1:s1258
RSF1:s604
FAM83H:s870
FAM83H:s914
FAM83H:s647
FAM83H:s998
NUP107:s37
b
phospho-proteins positively correlated with SV (350 features, p<0.05)
phospho-proteins for which total proteins were positively correlated with SV (681 features, p<0.05)
250
*
100
581
* overlap p<1E-30
AHNAK2:s2666
AHNAK2:s5428
ARHGEF1:s863
ARHGEF18:s760
ATE1:s169
CAVIN2:s293
CHAMP1:s108
CHAMP1:s204
CHAMP1:s204s214
CHAMP1:s382s386
CHAMP1:s386
CHAMP1:s405
CHAMP1:s416
CHAMP1:s445
CHAMP1:s476
CHAMP1:s542
CNP:s170
COBL:s269
COBL:s347
COBL:s815
CRYBG1:s329
CUX1:s663
DOCK7:s1383
EGFR:s946
EGFR:t648
ERBB2:s968
ERBB2:t671
FAM83H:s1003
FAM83H:s411
FAM83H:s870
FAM83H:s892
FAM83H:s903
FAM83H:s914
GCFC2:s174s180
GCFC2:s180
GCFC2:s96t97
GRB10:s104
IRS2:s560
IRS2:s594
IRS2:s915
IRS2:s973
ITPRID2:s668
ITPRID2:s767
LEMD3:s144
MAP2K2:t394
MCM6:s762
MORC2:s777s779
NCL:s67
NCL:t121
NCL:t76
NES:s1016
NES:s1496s1498
NES:s471
NES:s548
NES:s578
NES:s680
NES:s702
NES:s768
NES:s905
NES:s934
NES:t315
NES:t338
NFIA:s280s287
NFIA:s287
NOLC1:s573
NUP107:s37
NUP210:t1844
PAK1:s204
PPFIA1:s666
PTGES3:s117
RECQL5:s815
RHPN2:s639
RSF1:s1258
RSF1:s604
RSL1D1:s427
SEC62:t375
SENP2:s344
SNAP29:s163
SQSTM1:s272
SQSTM1:t269
SQSTM1:t269s272
SRRM1:s234
SRRM1:s708
SRRM1:t884s886
SRRM2:s1083
SRRM2:s1124
SRRM2:s1866s1869
SRRM2:s484s486
SRRM2:s950s952s954
SRRM2:s952s954
SRRM2:t1003
SRRM2:t2738s2740
STIM2:s261
STIM2:s669
TACC3:s434
TBC1D23:s507
TBC1D23:s520
TEX2:s538
TRIM28:s501
TRIM28:s697
phospho-proteins negatively correlated with SV (265 features, p<0.05)
phospho-proteins for which total proteins were negatively correlated with SV (299 features, p<0.05)
239
*
26
273
* overlap p<0.0001
C1orf52:s158
CD44:s706
CNN2:s166
DOCK10:s1251
DOCK10:s13
FAF1:s320
MICAL3:s1129
MICAL3:s1310
MICAL3:s1483
MPRIP:s619
NF1:s2494
NF1:s2522
PSIP1:t122s129
RB1:s249
RNF31:s315
SETX:s2639
STK17A:s28
TAOK1:s9
TPM1:s87
TSC1:s505
WDR7:s935
WIPF3:s281
XIAP:s406
c
increased with nearby breakpoint
decreased with nearby breakpoint
gene p-value by protein (1Mb region)
gene p-value by phospho-protein (1Mb region)
e-12
e-8
e-6
e-5
e-4
e-3
0.01
0.1
1
CHAMP1
EGFR
RSF1
XIAP
NUP107
GRB10
COBL
ERBB2
FAM83H
IRS2
CAVIN2
ITPRID2
MORC2
SRRM1
GCFC2
WDR7
FAF1
DOCK10
SNAP29
TSC1
NUP210
MAST4
NF1
WIPF3
SQSTM1
PPFIA1
TRIM28
ARHGEF1
NCL
AHNAK2
CRYBG1
NES
ATE1
PSIP1
RNF31
SRRM2
PLEKHG2
SUPT5H

Supplementary Figure 3. Genes with altered phospho-protein expression associated with nearby somatic SV breakpoints. (a) Heatmap of significance patterns for 200 phospho-proteins for which the corresponding total protein expression is associated with SV-altered expression (FDR<10%) for any genomic region window examined (involving SV breakpoints 100kb upstream of the gene, 100kb downstream of the gene, within the gene body, or 1MB upstream or downstream of the gene, as indicated). Red denotes a significant positive correlation; blue, significant negative correlation. Gene-level significance results at the total protein level are represented alongside results at the phospho-protein level. Proteins listed by name were significant at both total and phospho-protein levels for at least one genomic region window. SV-protein association p-values correct for cancer type and CNA by linear modeling. **(b)** Venn diagrams representing the overlaps between the genes with proteins correlated ($p<0.05$) with SV breakpoint at the total protein and phospho-protein levels. The left diagram is for positively correlated proteins; the right is for negatively correlated proteins. SV-expression association p-values based on 1Mb region and correct for cancer type and CNA by linear modeling. Significance of overlap by one-sided Fisher's exact test. **(c)** Significance of SV-impacted genes at the total protein level (involving breakpoints within 1Mb of the gene), as plotted (y-axis) versus the significance at the phospho-protein level (x-axis). "Cancer-related," by COSMIC[11]. SV-expression association p-values based on 1Mb region and correct for cancer type and CNA by linear modeling.

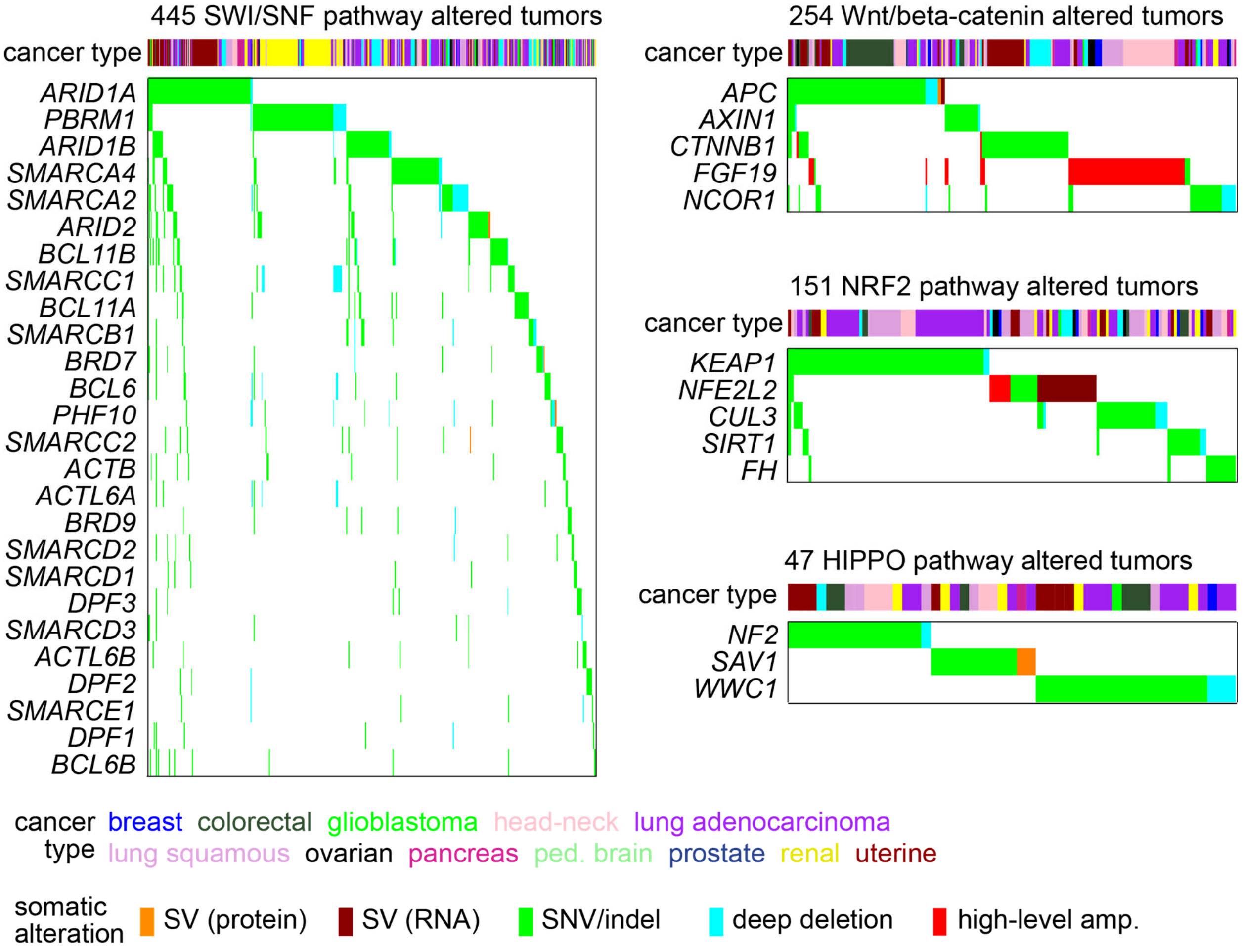

445 SWI/SNF pathway altered tumors
cancer type
ARID1A
PBRM1
ARID1B
SMARCA4
SMARCA2
ARID2
BCL11B
SMARCC1
BCL11A
SMARCB1
BRD7
BCL6
PHF10
SMARCC2
ACTB
ACTL6A
BRD9
SMARCD2
SMARCD1
DPF3
SMARCD3
ACTL6B
DPF2
SMARCE1
DPF1
BCL6B
254 Wnt/beta-catenin altered tumors
cancer type
APC
AXIN1
CTNNB1
FGF19
NCOR1
151 NRF2 pathway altered tumors
cancer type
KEAP1
NFE2L2
CUL3
SIRT1
FH
47 HIPPO pathway altered tumors
cancer type
NF2
SAV1
WWC1
cancer type
breast
colorectal
glioblastoma
head-neck
lung adenocarcinoma
lung squamous
ovarian
pancreas
ped. brain
prostate
renal
uterine
somatic alteration
SV (protein)
SV (RNA)
SNV/indel
deep deletion
high-level amp.

Supplementary Figure 4. Additional information regarding key oncogenic or tumor-suppressive pathways. For the pathways from Figure 4b that involved very few SV events with protein over-expression (i.e., pathways represented in Figure 4b but not in Figure 4c), somatic alteration events involving each gene included in the pathway are represented. SV events represent altered gene expression (by protein or alternatively by mRNA if protein data not available), defined for oncogenes as breakpoint falling with 1 Mb of gene and associated with expression >0.4 SD from the median for the given tumor, and defined for tumor suppressors as breakpoint falling within the gene body and expression < −0.4 SD. Events are colored according to the type of somatic alteration: gene fusion, SV with altered expression (by protein or alternatively by mRNA if protein data not available), SNV or indel (for oncogenes, SNV within hotspot residue; for tumor suppressor genes, SNV within hotspot residue or inactivating mutation by indel/nonsense/nonstop), and deep deletion or high-level amplification (respectively approximating total copy loss and copy levels more than 2× greater than that of wild-type, based on thresholded values).

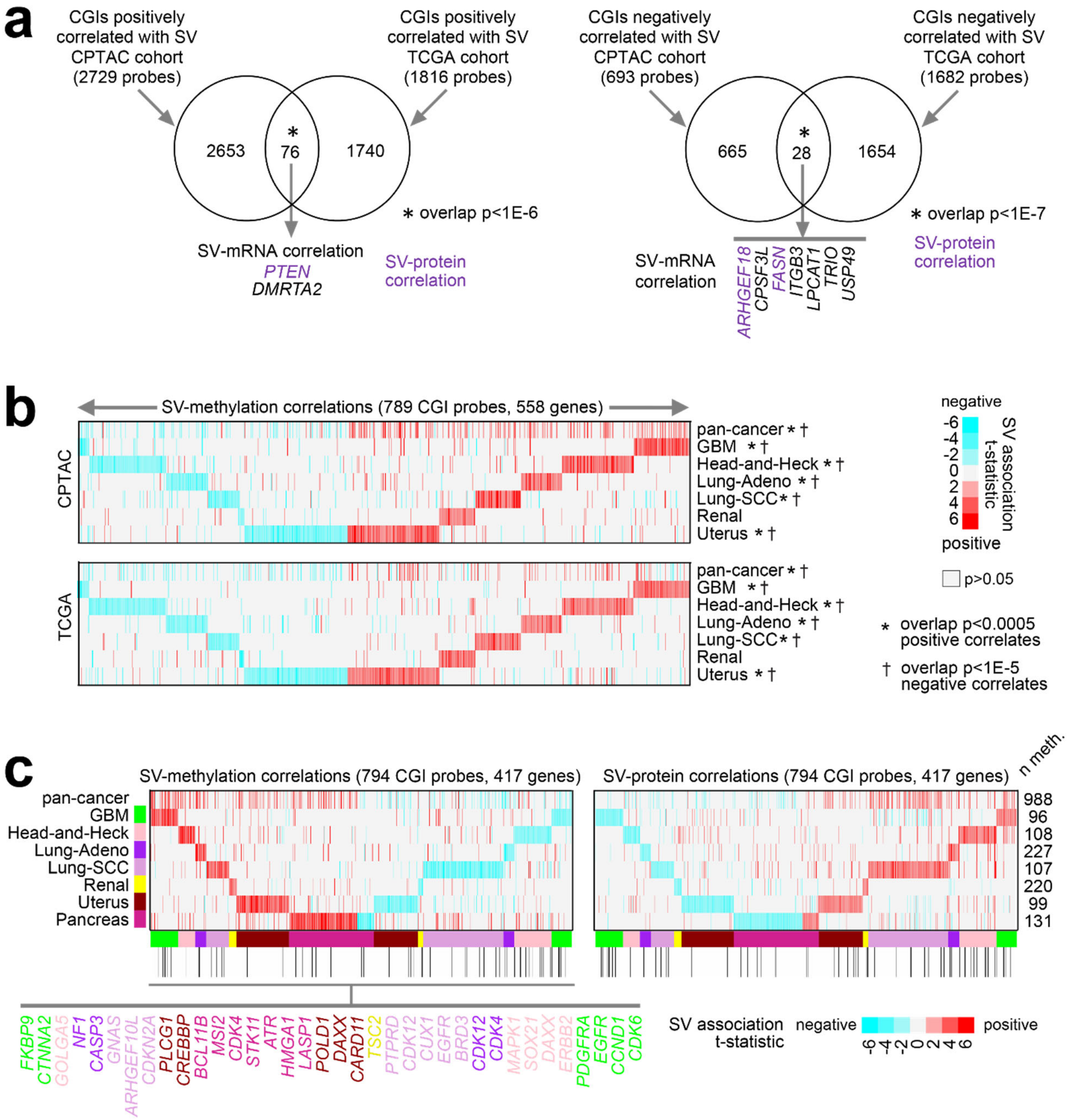
a
CGIs positively correlated with SV CPTAC cohort (2729 probes)
CGIs positively correlated with SV TCGA cohort (1816 probes)
2653
*
76
1740
* overlap p<1E-6
SV-mRNA correlation
PTEN
DMRTA2
SV-protein correlation
CGIs negatively correlated with SV CPTAC cohort (693 probes)
CGIs negatively correlated with SV TCGA cohort (1682 probes)
665
*
28
1654
* overlap p<1E-7
SV-mRNA correlation
ARHGEF18
CPSF3L
FASN
ITGB3
LPCAT1
TRIO
USP49
SV-protein correlation
b
SV-methylation correlations (789 CGI probes, 558 genes)
CPTAC
TCGA
pan-cancer * †
GBM * †
Head-and-Heck * †
Lung-Adeno * †
Lung-SCC * †
Renal
Uterus * †
negative
-6
-4
-2
0
2
4
6
positive
SV association t-statistic
p>0.05
* overlap p<0.0005 positive correlates
† overlap p<1E-5 negative correlates
c
SV-methylation correlations (794 CGI probes, 417 genes)
SV-protein correlations (794 CGI probes, 417 genes)
n meth.
pan-cancer 988
GBM 96
Head-and-Heck 108
Lung-Adeno 227
Lung-SCC 107
Renal 220
Uterus 99
Pancreas 131
FKBP9
CTNNA2
GOLGA5
NF1
CASP3
GNAS
ARHGEF10L
CDKN2A
PLCG1
CREBBP
BCL11B
MSI2
CDK4
STK11
ATR
HMGA1
LASP1
POLD1
DAXX
CARD11
TSC2
PTPRD
CDK12
CUX1
EGFR
BRD3
CDK12
CDK4
MAPK1
SOX21
DAXX
ERBB2
PDGFRA
EGFR
CCND1
CDK6
SV association t-statistic
negative
positive
-6
-4
-2
0
2
4
6

Supplementary Figure 5. Additional information on altered DNA methylation patterns associated with nearby somatic SV breakpoints. (a) Venn diagrams representing the overlaps between CGI methylation probes correlated ($p<0.01$) with SV breakpoint in the CPTAC compendium cohort (n=988 tumors) and the CGI probes correlated ($p<0.01$) with SV breakpoint in TCGA cohort (n = 1482 tumors)[10]. The left diagram is for positively correlated CGIs; the right diagram is for negatively correlated CGIs. P values by chi-square test. SV-methylation associations based on 1Mb region and correct for cancer type and CNA by linear modeling. Noted protein and mRNA correlations significant with one-sided $p<0.05$ (1Mb). **(b)** Between the CPTAC and TCGA cohorts, a set of 789 CGI probes (representing 558 genes) had CGI methylation significantly associated with nearby SV breakpoints for one or more individual cancer types for both cohorts ($p<0.05$ for each, with consistent direction). SV-expression associations are based on 1Mb region and correct for cancer type and CNA. For each cancer type, significant gene set overlap between positive or negative SV-expression correlates evaluated by one-sided Fisher's exact or chi-square tests. **(c)** In the CPTAC cohort, a set of 794 CGI probes (involving 417 genes) significantly associated with nearby SV breakpoints for one or more individual cancer types for both DNA methylation and protein expression of the associated gene ($p<0.05$ for each, with consistent direction). The left panel represents SV-methylation associations; the right panel represents SV-protein associations. SV-expression associations are based on 1Mb region and correct for cancer type and CNA. Genes listed by name are COSMIC genes, with name coloring corresponding to the associated cancer type. For parts b and c, CGI probes featured were filtered for those probes for which at least one tumor has an SV breakpoint within 1MB of the associated genes, where the absolute associated DNA methylation beta-value change from the median across all tumors was greater than 0.2.

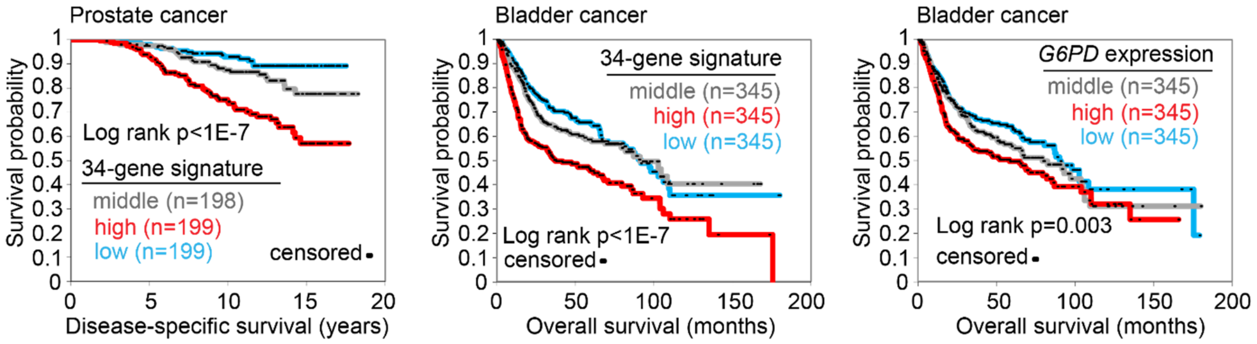

Supplementary Figure 6. Somatic SV-altered proteins involving patient survival in prostate cancer and bladder cancer. Association of the 34-gene expression signature from Figure 7a with patient survival across prostate cancer (n=596)[12] and bladder cancer (n=1,035)[13-17] expression datasets. The bladder cancer dataset represents a compendium of five separate datasets, assembled using the same approaches used previously in assembling the lung adenocarcinoma compendium in Figure 7[2,18]. Also shown is the association of *G6PD* mRNA with worse outcome in the bladder cancer compendium dataset. *G6PD* was not represented in the prostate cancer dataset. P-values by log-rank test.

Supplementary References

1. Wilkerson, M. & Hayes, D. ConsensusClusterPlus: a class discovery tool with confidence assessments and item tracking. *Bioinformatics* **26**, 1572-1573 (2010).
2. Monsivais, D., *et al.* Mass-spectrometry-based proteomic correlates of grade and stage reveal pathways and kinases associated with aggressive human cancers. *Oncogene* **40**, 2081-2095 (2021).
3. Chen, F., Chandrashekar, D., Varambally, S. & Creighton, C. Pan-cancer molecular subtypes revealed by mass-spectrometry-based proteomic characterization of more than 500 human cancers. *Nat Commun* **10**, 5679 (2019).
4. Zhang, Y., Chen, F., Chandrashekar, D., Varambally, S. & Creighton, C. Proteogenomic characterization of 2002 human cancers reveals pan-cancer molecular subtypes and associated pathways. *Nat Commun* **13**, 2669 (2022).
5. Zhang, Y., Chen, F. & Creighton, C. Pan-cancer molecular subtypes of metastasis reveal distinct and evolving transcriptional programs. *Cell Rep Med* **4**, 100932 (2023).
6. Chen, F., *et al.* Pan-cancer molecular classes transcending tumor lineage across 32 cancer types, multiple data platforms, and over 10,000 cases. *Clin Cancer Res.* **24**, 2182-2193 (2018).
7. Chen, F., *et al.* Pan-urologic cancer genomic subtypes that transcend tissue of origin. *Nat Commun* **8**, 199 (2017).
8. Gillette, M., *et al.* Proteogenomic Characterization Reveals Therapeutic Vulnerabilities in Lung Adenocarcinoma. *Cell* **182**, 200-225 (2020).
9. Soltis, A., *et al.* Proteogenomic analysis of lung adenocarcinoma reveals tumor heterogeneity, survival determinants, and therapeutically relevant pathways. *Cell Rep Med* **3**, 100819 (2022).
10. Zhang, Y., *et al.* Global impact of somatic structural variation on the DNA methylome of human cancers. *Genome biology* **20**, 209 (2019).
11. Forbes, S., *et al.* COSMIC: somatic cancer genetics at high-resolution. *Nucleic Acids Res* **45**, D777-D783 (2017).
12. Nakagawa, T., *et al.* A tissue biomarker panel predicting systemic progression after PSA recurrence post-definitive prostate cancer therapy. *PloS one* **3**, e2318 (2008).
13. Cancer_Genome_Atlas_Research_Network. Comprehensive molecular characterization of urothelial bladder carcinoma. *Nature* **507**, 315-322 (2014).
14. Kim, W., *et al.* Predictive value of progression-related gene classifier in primary non-muscle invasive bladder cancer. *Mol Cancer* **9**, 3 (2010).
15. Riester, M., *et al.* Combination of a novel gene expression signature with a clinical nomogram improves the prediction of survival in high-risk bladder cancer. *Clin Cancer Res.* **18**, 1323-1333 (2012).
16. Sjödahl, G., *et al.* A molecular taxonomy for urothelial carcinoma. *Clin Cancer Res.* **18**, 3377-3386 (2012).
17. Choi, W., *et al.* Identification of distinct basal and luminal subtypes of muscle-invasive bladder cancer with different sensitivities to frontline chemotherapy. *Cancer Cell* **25**, 152-165 (2014).
18. Chen, F., *et al.* Multiplatform-based Molecular Subtypes of Non-Small Cell Lung Cancer. *Oncogene* **36**, 1384-1393 (2016).
