# Supplementary material for: Global impact of somatic structural variation on the cancer proteome
Source: Nat Commun. 2023 Sep 13;14:5637. doi: 10.1038/s41467-023-41374-8 (PMC10499989; doi:10.1038/s41467-023-41374-8)
Supplement: Supplementary file 3 — Description of Additional Supplementary Files [file 41467_2023_41374_MOESM3_ESM.pdf]

Supplementary Data Files

Supplementary Data 1. Tumor-level sample annotation table. Provided as an Excel file. By tumor, sample annotation, pathway-level genetic and genomic alterations, and other information are provided for the SV-expression compendium dataset. Also included is a list of datasets incorporated into the study and a list of datasets and samples used for the gene-level breakpoint pattern survival analysis.

Supplementary Data 2. Gene-level associations between protein expression and nearby SV breakpoint. Provided as an Excel file. Complete set of gene-level associations between protein expression and nearby somatic SV breakpoint in the compendium cohort, according to region examined (e.g., 0–100 kb upstream, 0–100 kb downstream, within the gene body, or 1 Mb upstream or downstream). Results are included for both total protein features and phosphoprotein features. Gene Ontology (GO) term enrichment and associated genes are provided for the top significant proteins (1Mb region).

Supplementary Data 3. Gene-level associations between mRNA expression and nearby SV breakpoint. Provided as an Excel file. Complete set of gene-level associations between mRNA expression and nearby somatic SV breakpoint in the compendium cohort, according to region examined (e.g., 0–100 kb upstream, 0–100 kb downstream, within the gene body, or 1 Mb upstream or downstream). mRNA results are provided for both the full mRNA dataset, including expression values that may not be represented in the protein dataset (e.g., protein expression not detected for a particular gene in a particular tumor), and for a filtered mRNA dataset, with any expression data values not represented in the protein dataset removed. Gene Ontology (GO) term enrichment and associated genes are provided for the top significant mRNAs (1Mb region).

Supplementary Data 4. Gene fusion predictions as made by RNA-seq chimeric reads with WGS and expression support in the compendium cohort. Provided as an Excel file.

Supplementary Data 5. SVs associated with genes of interest, with associated expression. Provided as an Excel file. Data provided for genes with associated SV events highlighted in the figures, e.g., for genes *AKR1C1*, *IGF2* (Figure 1), *KRAS*, *EGFR*, *FGFR2*, *FGFR3*, *NF1*, *ERBB2*, *E2F3*, *CCND1*, *CCNE1*, *CDK4*, *RB1*, *CDKN2A*, *TERT*, *MYC*, *MYB*, *MYCN*, *PTEN*, *AKT1*, *STK11*, *PIK3CA* (Figure 4), *ANO1*, *NID2* (Figure 5), *G6PD* (Figure 7).

Supplementary Data 6. Probe-level CpG Island (CGI) associations between DNA methylation and nearby SV breakpoint. Provided as an Excel file. Complete set of CGI probe-level associations between methylation and nearby somatic SV breakpoint in the compendium cohort (CPTAC tumors), according to region examined (e.g., 0–100 kb upstream, 0–100 kb downstream, within the gene body, or 1 Mb upstream or downstream).

Supplementary Data 7. Top gene-level associations between expression or methylation and nearby SV breakpoint for each cancer type. Provided as an Excel file. Data for top genes or CGI probes showing expression (protein or mRNA) or DNA methylation associations

with SV breakpoints for one or more tissue- or histology-based cancer types within the compendium cohort, representing results highlighted in the figures.

Supplementary Data 8. Somatic SV associations with translocated enhancers, translocated retrotransposons, and rearrangement of regions with high or low methylation. Provided as an Excel file. Results include the subset of somatic SV breakpoint associations with overexpression or underexpression and involving the translocation of an enhancer within 0.5 Mb of the gene (and closer to the gene as compared to any enhancer involved in the unaltered region). Results also include the subset of somatic SV breakpoint associations with overexpression or underexpression and involving the translocation of a LINE or SINE within 20kb of the gene (and closer to the gene as compared to any enhancer involved in the unaltered region, respectively). Results also include the SV events involving the rearrangement of a region of low methylation (average methylation beta difference < -0.1), with a corresponding decrease in methylation and increased protein expression observed.

Supplementary Data 9. Gene-level correlations with patient overall survival. Provided as an Excel file. By stratified Cox, correcting for cancer type, associations with both nearby SV breakpoints and expression are provided. For expression, positive Cox beta indicates that higher expression is associated with worse outcome; negative Cox beta, associated with better outcome. For SV breakpoints, positive Cox beta indicates that nearby breakpoints are associated with worse outcome; negative Cox beta, associated with better outcome.

Supplementary Data 10. Cancer cell line associations. Provided as an Excel file. Results include gene-level associations between expression (protein and mRNA) and nearby SV breakpoint, with DepMap-related results.
