# Supplementary material for: Global impact of somatic structural variation on the cancer proteome
Source: Nat Commun. 2023 Sep 13;14:5637. doi: 10.1038/s41467-023-41374-8 (PMC10499989; doi:10.1038/s41467-023-41374-8)
Supplement: Supplementary file 14 — Reporting Summary [file 41467_2023_41374_MOESM14_ESM.pdf]

nature research

Corresponding author(s): Chad Creighton
Last updated by author(s): Jul 25, 2023

Reporting Summary

Nature Research wishes to improve the reproducibility of the work that we publish. This form provides structure for consistency and transparency in reporting. For further information on Nature Research policies, see Authors & Referees and the Editorial Policy Checklist.

Statistics

For all statistical analyses, confirm that the following items are present in the figure legend, table legend, main text, or Methods section.

n/a	Confirmed	
☐	☒	The exact sample size (*n*) for each experimental group/condition, given as a discrete number and unit of measurement
☐	☒	A statement on whether measurements were taken from distinct samples or whether the same sample was measured repeatedly
☐	☒	The statistical test(s) used AND whether they are one- or two-sided<br>*Only common tests should be described solely by name; describe more complex techniques in the Methods section.*
☐	☒	A description of all covariates tested
☐	☒	A description of any assumptions or corrections, such as tests of normality and adjustment for multiple comparisons
☐	☒	A full description of the statistical parameters including central tendency (e.g. means) or other basic estimates (e.g. regression coefficient) AND variation (e.g. standard deviation) or associated estimates of uncertainty (e.g. confidence intervals)
☐	☒	For null hypothesis testing, the test statistic (e.g. *F*, *t*, *r*) with confidence intervals, effect sizes, degrees of freedom and *P* value noted<br>*Give P values as exact values whenever suitable.*
☐	☒	For Bayesian analysis, information on the choice of priors and Markov chain Monte Carlo settings
☐	☒	For hierarchical and complex designs, identification of the appropriate level for tests and full reporting of outcomes
☐	☒	Estimates of effect sizes (e.g. Cohen's *d*, Pearson's *r*), indicating how they were calculated

Our web collection on statistics for biologists contains articles on many of the points above.

Software and code

Policy information about availability of computer code

Data collection	The results here are based upon data from public respositories. No specialized in-house software was used for data collection. Microsoft Excel was used to transform expression values and to concatenate datasets as described in Methods. The compiled molecular datasets used for the SV breakpoint-expression linear modeling are deposited into FigShare.
Data analysis	No unpublished specialized code was written for this study. Using SVExpress [PMID: 33743584], we defined genes with altered expression (by protein or mRNA) associated with nearby somatic SV breakpoints. SVExpress is freely available for academic or commercial use at https://github.com/chadcreighton/SVExpress . SVExpress is implemented as a set of Excel macros and R code. All source code (R and Visual Basic for Applications) is available. Visualization using heat maps was performed using both JavaTreeview (version 1.1.6r4) and matrix2png (version 1.2.1). SigTerms is available at Sourceforge [http://sigterms.sourceforge.net/]

For manuscripts utilizing custom algorithms or software that are central to the research but not yet described in published literature, software must be made available to editors/reviewers. We strongly encourage code deposition in a community repository (e.g. GitHub). See the Nature Research guidelines for submitting code & software for further information.

Data

Policy information about availability of data

All manuscripts must include a data availability statement. This statement should provide the following information, where applicable:
- Accession codes, unique identifiers, or web links for publicly available datasets
- A list of figures that have associated raw data
- A description of any restrictions on data availability

All data used in this study are publicly available. Proteomics data are available for CPTAC, CBTN, and ICPC studies at the Proteomic Data Commons [https://pdc.cancer.gov/]. For CPTAC, APOLLO, and TCGA studies, transcriptome data, copy number data, and small somatic mutation data are available at the Genomic Data Commons [https://gdc.cancer.gov/]. CBTN genomic and transcriptomic data are available via Kids First Data Resource Portal and Cavatica (https://cbtn.org/). Cancer Cell Line Encyclopedia (CCLE) datasets are available from the CCLE website [http://www.broadinstitute.org/ccle]. Genomics of Drug Sensitivity in Cancer (GDSC) cell line data are available from the Sanger Institute web site [https://www.sanger.ac.uk/tool/gdsc-genomics-drug-sensitivity-cancer/]. For other published

studies, molecular data availability information is provided in the associated publication. The compendium datasets of molecular profiles for total protein, CNA, and mRNA—compiled as part of our study—are available through FigShare. Any remaining data are available within the Article, Supplementary Information, or from the authors. Source data are provided with this paper. (There are no direct hyperlinks for CPTAC and other cohorts with data in the GDC data portal.)

Field-specific reporting

Please select the one below that is the best fit for your research. If you are not sure, read the appropriate sections before making your selection.

☒ Life sciences ☐ Behavioural & social sciences ☐ Ecological, evolutionary & environmental sciences

For a reference copy of the document with all sections, see nature.com/documents/nr-reporting-summary-flat.pdf

Life sciences study design

All studies must disclose on these points even when the disclosure is negative.

Sample size	Sample size used in the study was based on all available data in the public domain with both WGS and proteomic profiling data. No sample calculations were performed, though the number of tumors analyzed is comparable to our previous WGS-expression study as part of PCAWG involving 1220 tumors, as well as (for most proteins) exceeding the numbers involved in our previous study of the POG570 dataset involving 570 tumors. Combined WGS and mass spectrometry-based proteomic profiling was compiled for 1307 tumors in total, representing 1290 patients (Table S1). The cancer types represented in the proteomics compendium dataset were the following: Breast Invasive Carcinoma (n=18 tumors with proteomics data), Colorectal Adenocarcinoma (n=46), Glioblastoma (n=98), Head and Neck Squamous Cell Carcinoma (n=108), Lung Adenocarcinoma (n=197), Lung Squamous Cell Carcinoma (n=108), Ovarian Serous Cystadenocarcinoma (n=15), Pancreatic Ductal Adenocarcinoma (n=136), Pediatric Brain Tumors (n=219), Prostate Adenocarcinoma (n=47), Renal Cell Carcinoma (n=219), and Uterine Corpus Endometrial Carcinoma (n=99). Combined WGS and RNA-seq profiling was compiled for 1413 tumors in total, of which 1294 had proteomic data and of which 988 had DNA methylation data (Illumina MethylationEPIC platform) not analyzed by our group previously. For 118 lung adenocarcinomas in CPTAC Confirmatory cohort with combined WGS, RNA-seq, and DNA methylation data, no proteomics data were made publicly available at the time of our study.
Data exclusions	No data were excluded from the analysis.
Replication	Our present study involving a combined WGS and expression compendium dataset confirmed the overall observations on SV-altered expression patterns made previously using PCAWG, TCGA, CBTN, and POG570 WGS datasets. These observations included hundreds of genes with altered expression recurrently associated with nearby somatic SV breakpoints, DNA methylation changes associated with SV breakpoints, and mechanisms involving altered cis-regulation including enhancer hijacking and translocation of retrotransposons. Where possible, we confirmed broad and statistically significant overlap in results between our present cohort and results from the previous cohorts examined. Genes significant for one cohort but not the other may be attributable to several factors, involving differences in tumor type representation, false positives, and false negatives. Gene-associated SV breakpoint events may be sparse for any gene; in other words, for a given gene, on the order of just 1-3% of tumors in a cohort may have SV breakpoints within 1Mb of that gene after amplification events are excluded (see Figure 2c). Given this sparseness in amplification-independent SV breakpoint patterns, it would not be surprising for a gene to have significant SV-expression associations in some but not all cohorts examined. In addition, here, we could identify SV breakpoint patterns and corresponding aberrantly expression genes associated with patient survival and genes with nearby SV breakpoints associated with increased cell dependency in cancer cell lines. We did not run controlled experiments involving limited numbers of cell cultures or of mice.
Randomization	Not applicable, no experimental data. We did not carry out a controlled experimental data whereby subjects or mice were assigned to different experimental groups.
Blinding	Blinding not relevant, as samples were not allocated to experimental groups.

Reporting for specific materials, systems and methods

We require information from authors about some types of materials, experimental systems and methods used in many studies. Here, indicate whether each material, system or method listed is relevant to your study. If you are not sure if a list item applies to your research, read the appropriate section before selecting a response.

Materials & experimental systems

n/a	Involved in the study	
☒	☐	Antibodies
☒	☐	Eukaryotic cell lines
☒	☐	Palaeontology
☒	☐	Animals and other organisms
☒	☐	Human research participants
☒	☐	Clinical data

Methods

n/a	Involved in the study	
☒	☐	ChIP-seq
☒	☐	Flow cytometry
☒	☐	MRI-based neuroimaging
